# Investigating shared decision-making during the use of a digital health tool for physical activity planning in cardiac rehabilitation

**DOI:** 10.3389/fdgth.2023.1324488

**Published:** 2024-01-04

**Authors:** Daniela Wurhofer, Julia Neunteufel, Eva-Maria Strumegger, Isabel Höppchen, Barbara Mayr, Andreas Egger, Mahdi Sareban, Bernhard Reich, Michael Neudorfer, Josef Niebauer, Jan David Smeddinck, Stefan Tino Kulnik

**Affiliations:** ^1^ Ludwig Boltzmann Institute for Digital Health and Prevention, Salzburg, Austria; ^2^ University Institute of Sports Medicine, Prevention and Rehabilitation and Research Institute of Molecular Sports Medicine and Rehabilitation, Paracelsus Medical University, Salzburg, Austria

**Keywords:** mHealth, mobile health, digital health, shared decision-making, cardiac rehabilitation, physical activity, exercise planning, behaviour change

## Abstract

**Background:**

Shared decision making (SDM) between healthcare professionals and persons with CVD can have a positive impact on motivation, adherence, or sustainability regarding long-term goals and integration of cardiovascular disease (CVD) rehabilitation in the everyday lives of persons with CVD. SDM can foster the transition between regular heart-healthy activity at rehabilitation facilities and more independent activity at home, but it is often challenging to implement SDM given limited time and resources, e.g., in the daily practice of rehabilitation. Digital tools can help but must be appropriately tailored for situated use and user needs.

**Objective:**

We aimed to (1) describe in how far SDM is manifested in the situated context when using a digital tool developed by our group, and, based on that, (2) reflect on how digital health tools can be designed to facilitate and improve the SDM process.

**Methods:**

In the context of a field study, we investigated how SDM is already naturally applied and manifested when using a digital tool for joint physical activity planning in cardiac rehabilitation in clinical practice. In a two-week qualitative study, we collected data on expectations, experiences and interactions during the use of a digital health tool by seven persons with CVD and five healthcare professionals. Data was collected by means of observations, interviews, questionnaires and a self-reported diary, and analysed with a particular focus on episodes related to SDM.

**Results:**

We found that SDM was manifested in the situated context to limited extent. For example, we identified high improvement potential for more structured goal-setting and more explicit consideration of preferences and routines. Based on mapping our findings to temporal phases where SDM can be adopted, we highlight implications for design to further support SDM in clinical practice. We consider this as “SDM supportive design in digital health apps,” suggesting for example step-by-step guidance to be used during the actual consultation.

**Conclusion:**

This study contributes to further understanding and integration of SDM in digital health tools with a focus on rehabilitation, to empower and support both persons with CVD and healthcare professionals.

## 1 Introduction

Insufficient physical activity (PA) is a major global public health problem (1). Regular PA (2, 3) is associated with many positive effects on health (4) and therefore an important measure in prevention and rehabilitation of chronic diseases (5).

A major challenge with regard to regular PA is sustainable behaviour change on an individual level (6, 7). Digital health interventions can offer valuable support with regard to adherence, motivation and sustainability (8–11). However, engagement with novel digital tools, such as health apps, does not occur in isolation; but often it is healthcare professionals who introduce persons with CVD to digital tools and encourage their use. In this context, digital tools may provide an additional valuable contribution to the communication between healthcare professionals and persons with CVD, by informing and facilitating this interaction (12).

Patients are increasingly seen as active partners in the management of their healthcare (13). This is reflected, for example, in the increasing prevalence of Shared Decision-Making (SDM) in clinical encounters, denoting “an approach where clinicians and patients share the best available evidence when faced with the task of making decisions, and where patients are supported to consider options, to achieve informed preferences” (14). Integrating SDM in digital tools to empower persons with CVD, support self-efficacy, and promote long-term behaviour change could offer new potential as shown for example by Bonneux et al. (15, 16), Reese et al. (17) and Cao et al. (18). Although some initial research has explored this topic (19), more research is needed about how digital health tools could support SDM. In particular, more detailed information is needed regarding where within the process of SDM for PA in rehabilitation additional (digital) support could be beneficial.

In the context of promoting sustainable PA for persons with CVD with cardiovascular disease (CVD), we have developed a digital tool (aktivplan) that supports healthcare professionals and persons with CVD to jointly plan and document a regular heart-healthy PA schedule (20). In this paper, we analyse the application of this tool in practice and discuss how it could be adapted to further support SDM. Specifically, we aimed to (1) describe in how far SDM is manifested in the situated context when using the digital tool, and, based on that, (2) reflect on how digital health tools can be designed to facilitate and improve the SDM process.

With our work, we contribute design recommendations for SDM in digital health tools that could be beneficial to persons with CVD and healthcare professionals in the field of CVD prevention and rehabilitation. Beyond that, our insights provide a starting point for better SDM integration in digital health tools for prevention and rehabilitation in general.

## 2 Methods

### 2.1 Study design

Patients and healthcare professionals conducted PA consultation sessions at which an exercise training plan was discussed and agreed upon. We used a mixed-methods research design collecting data based on the following materials and measures:

#### 2.1.1 Non-participant observation with a focus on SDM

The Observing Patient Involvement in Decision Making (OPTION, (21)) and Rochester Participatory Decision-Making (RPAD, (22)) rating scales were used to conduct non-participant observations with a focus on SDM. These scales provide key statements on observable behavior that promotes SDM. The observer reviewed video recordings of all PA consultation sessions and rated each item according to the extent to which a certain behavior was demonstrated in each session.

#### 2.1.2 Semi-structured interviews

Semi-structured interviews were conducted separately with with persons with CVD and healthcare professionals. The interview guides were developed in an iterative process: Initial interview questions were suggested by the study lead, then discussed and refined within a 1-hour workshop setting with three other experts in digital health, and finally piloted with representatives of the participant groups in order to ensure comprehensibility and adequate duration.

Two semi-structured interviews were conducted with each person with CVD. The first interview was conducted immediately after the PA consultation session. The interview guide included eight questions focusing on expectations and previous experiences regarding digital health tools and exercise training; expectations about the upcoming use of aktivplan; and the perception of the rapport with the healthcare professional and alignment of the exercise training plan with their personal preferences. The second interview was conducted after the 14-day usage phase of the aktivplan app. This interview guide consisted of eight questions with a focus on retrospective reflections about aktivplan, i.e., experiences and potential issues when using the aktivplan app in everyday life, suggestions for improvement, and the feeling of being connected to the healthcare professional via the aktivplan app.

One semi-structured interview was conducted with each healthcare professional shortly after the PA consultation session. The interview guide included seven questions covering healthcare professionals’ expectations, experiences, and possible difficulties when using a digital tool for planning exercise training in the context of CVD rehabilitation.

#### 2.1.3 Diary

A pen and paper diary was designed to collect daily self-reported data from persons with CVD. The study lead conducted a 90-min workshop with three experts in digital health to formulate potential diary questions. A first version of the diary was then developed, discussed further, and finalised in consultation with the digital health experts. A diary was chosen for data collection to obtain continuous insights over a 14-day period about technology interaction and other aspects of use. The main topics addressed were usage (frequency), usability, user experience and acceptance of the aktivplan app, relationship with the healthcare professional (mediated by aktivplan), as well as planning and documentation of training activities via aktivplan. The diary was designed to be filled in daily. Each day, three to five questions were provided in relation to participants’ training activities and prototype usage experience, mixing quantitative questions (e.g., indicating daily motivation for exercise training on a Likert-scale) and qualitative open-ended questions (e.g., describing one’s favourite experience with the app). Completing the diary could take approximately between five and 15 min per day, depending on how thoroughly open-ended questions were answered.

### 2.2 Participants

Participants of the study involved both CVD rehabilitation persons with CVD and healthcare professionals.

#### 2.2.1 Persons with CVD

We recruited current patients of a rehabilitation facility in Salzburg, Austria. To be included in the study, participants had to be 18 years and older and affected by CVD with current or previous participation in medical exercise therapy, i.e., persons with CVD in cardiac rehabilitation. For details on inclusion and exclusion criteria see Supplementary Data.

#### 2.2.2 Healthcare professionals

We recruited healthcare professionals including doctors and training therapists who regularly plan and monitor exercise training of persons with CVD.

### 2.3 aktivplan app

The app used in the study represents a planning tool for heart-healthy PA, which is used by healthcare professionals together with their patients. In contrast to common health apps, users of aktivplan are persons with CVD, and it was a deliberate design decision to incorporate healthcare professionals in the app and in its intended use (e.g., by having meetings with healthcare professionals in a clinical setting or by representing patient’s allocated healthcare professionals in the app).

**Figure 1:**
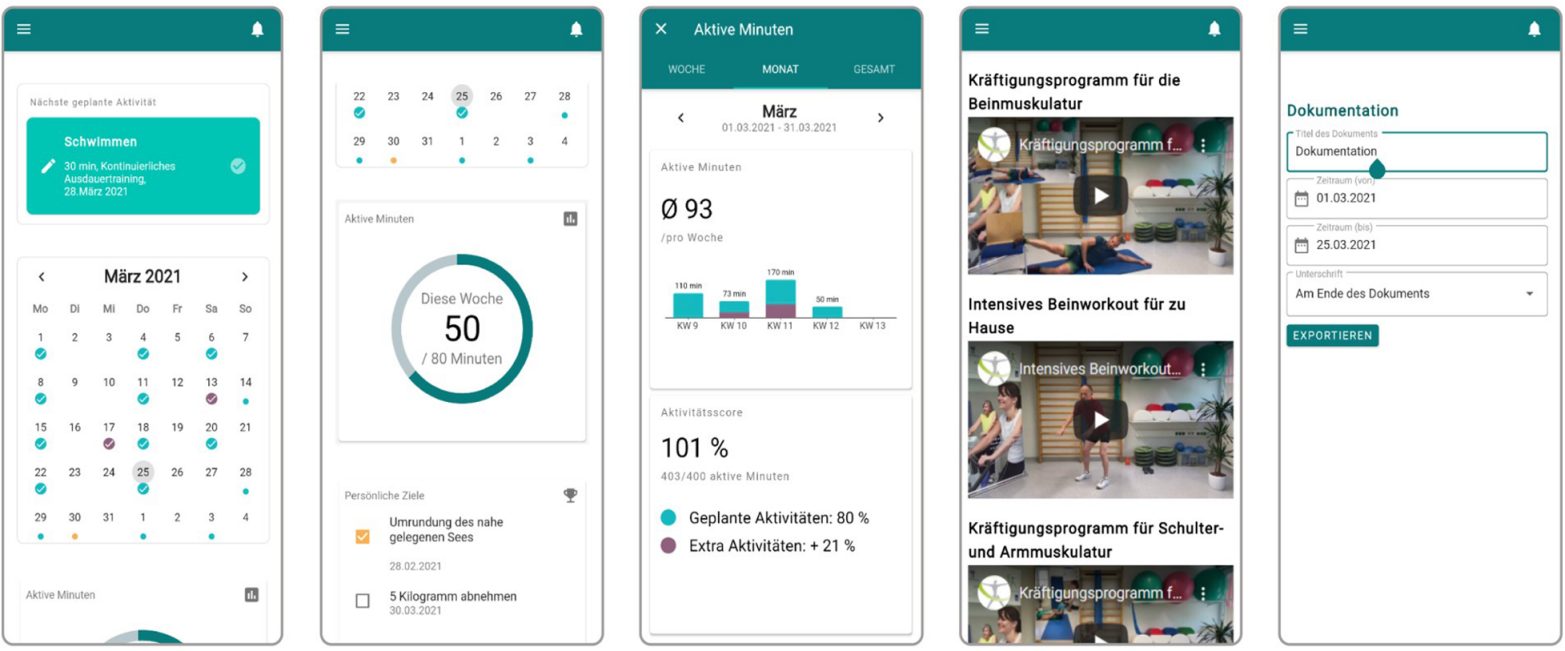
Main functionalities of aktivplan: (1) calendar with training plan, (2) overview of active minutes per week and personal goals, (3) monthly overview of active minutes, (4) videos with workouts, (5) export and documentation functionality.

The development of the aktivplan app was conceived as an explorative and iterative, user-centered design process involving healthcare professionals, persons with CVD and researchers (23, 24, 20). As shown in Figure 1, its main functions for persons with CVD include a calendar with an exercise training plan, which is established together with a healthcare professional and entered into the app by the healthcare professional; an overview of active minutes per week, indicating the progress of the current week, as well as a list of personal goals set together with the healthcare professional; an overview of active minutes per month showing the monthly progress; videos with workouts that can be performed at home; and a functionality to export and document the progress for healthcare professionals or health insurances. Overall, aktivplan is a tool that allows healthcare professionals and their patients to jointly set up, monitor, and regularly review a personalized heart-healthy PA plan. Patients are involved and guided by healthcare professionals in selecting exercises and activities they enjoy and defining personally meaningful goals. Through the app interface (intended for patients), patients can conveniently access their plan on a calendar, log, adjust or add activities, review their performance, and access a library of resources such as exercise videos. At follow-up appointments with their healthcare professional, patients can review their documented performance and discuss the plan going forward. The aktivplan app is intended to support a longer-term one-to-one relationship between a healthcare professional and patients. Healthcare professionals access a separate version of the app (web interface) and are supported in providing personalized exercise prescription as well as ongoing review and optimization of their patients’ performance. The exercise training plan is entered into the app by the healthcare professional. Through the web interface, healthcare professionals can conveniently view the patients’ activity logs. Activity logs can be exported and printed, to be filed in medical records, to provide documentation to health insurances, and to be used for joint review and further planning with patients at follow-up appointments. Direct communication between healthcare professionals and patients is not supported via the tool; rather, it is suggested that the exercise training plan should be reviewed periodically in follow-up consultation sessions, and that intermittent communication takes place using the healthcare professionals preferred and available channels such as email or telephone.

### 2.4 Study set-up & procedure

**Figure 2:**
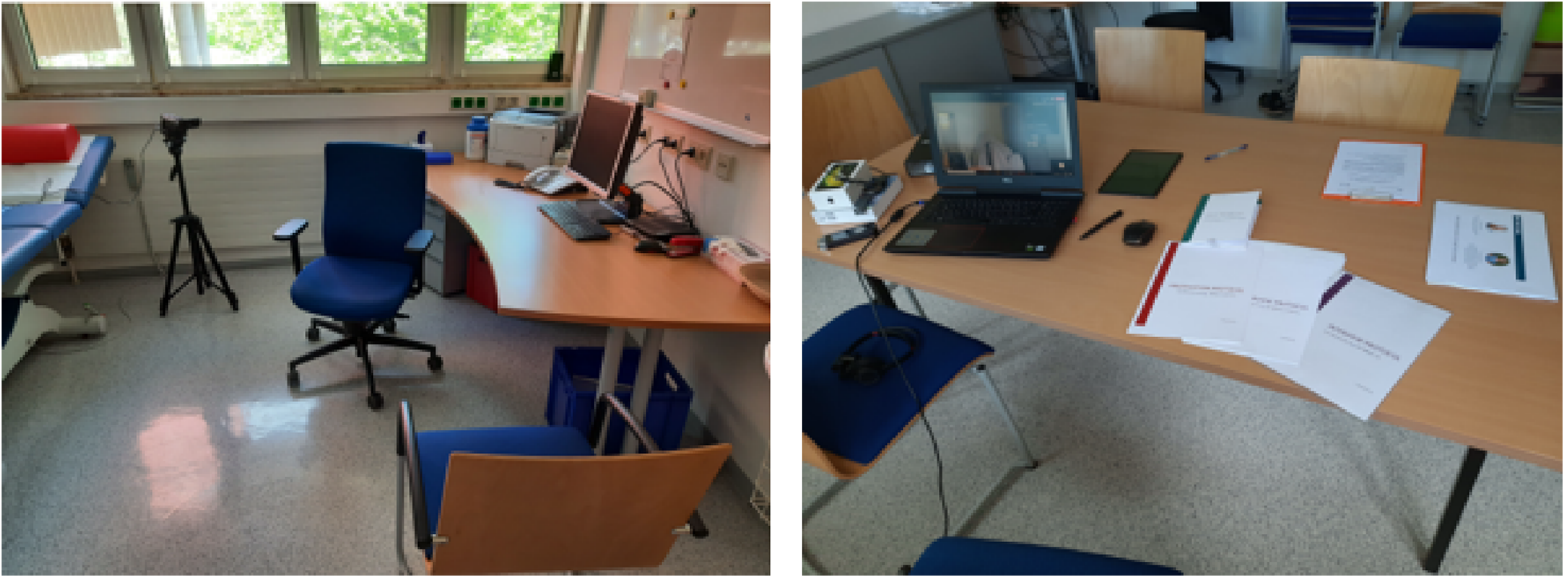
Room where the PA consultation session took place (left); room where interviews and observations were conducted (right).

All seven PA consultation sessions and interviews took place in a quiet and comfortable atmosphere (see Figure 2) at the affiliated rehabilitation facility during May and July 2021. The sessions and interviews lasted between 45 and 90 min each and were video- and/or audio-recorded based on prior informed consent. Before the start of data collection, healthcare professionals received a 30-min introductory session about the aktivplan app and its basic functionalities, including brief guidance on how to use the app during the PA consultation session. Healthcare professionals were provided with a two-page print-out that visualised the most important steps when using the app to establish an exercise training plan. Additionally, the overall aim of the study was explained, and the healthcare professionals had an opportunity to ask questions.

Regarding the study procedure, we distinguished between the introductory phase, the usage phase and the final phase.

The main aim of the **introductory phase** was to conduct the PA consultation session between healthcare professional and person with CVD, and to provide the aktivplan app to the person with CVD. First, the researcher informed the person with CVD about the study and obtained their consent. Then, the PA consultation session took place, aiming to establish an individual exercise training plan for the person with CVD for the forthcoming weeks. Healthcare professionals were instructed to base this on data from the person’s exercise capacity assessment (ergometry) as well as their individual preferences and routines. The planning was to be conducted by using the provided aktivplan tool on the healthcare professional’s desktop computer. Healthcare professionals explained the app and informed about data usage via the app; discussed routines and preferences with regard to PA with the person with CVD; and defined PA goals and an exercise training plan which were documented in the app. Healthcare professionals then asked the person with CVD to carry out their exercise training plan during the following two weeks. After the PA consultation session, the researcher interviewed the person with CVD, focusing on expectations and previous experiences regarding digital health tools and exercise training. Finally, the researcher helped the person with CVD to register and download the aktivplan app to ensure that everything was working properly before starting with the usage phase. After that, the researcher interviewed the healthcare professional regarding expectations, experiences and possible difficulties when using a digital tool for planning exercise training in the context of CVD rehabilitation (see Section 2.1.2).

**Figure 3:**
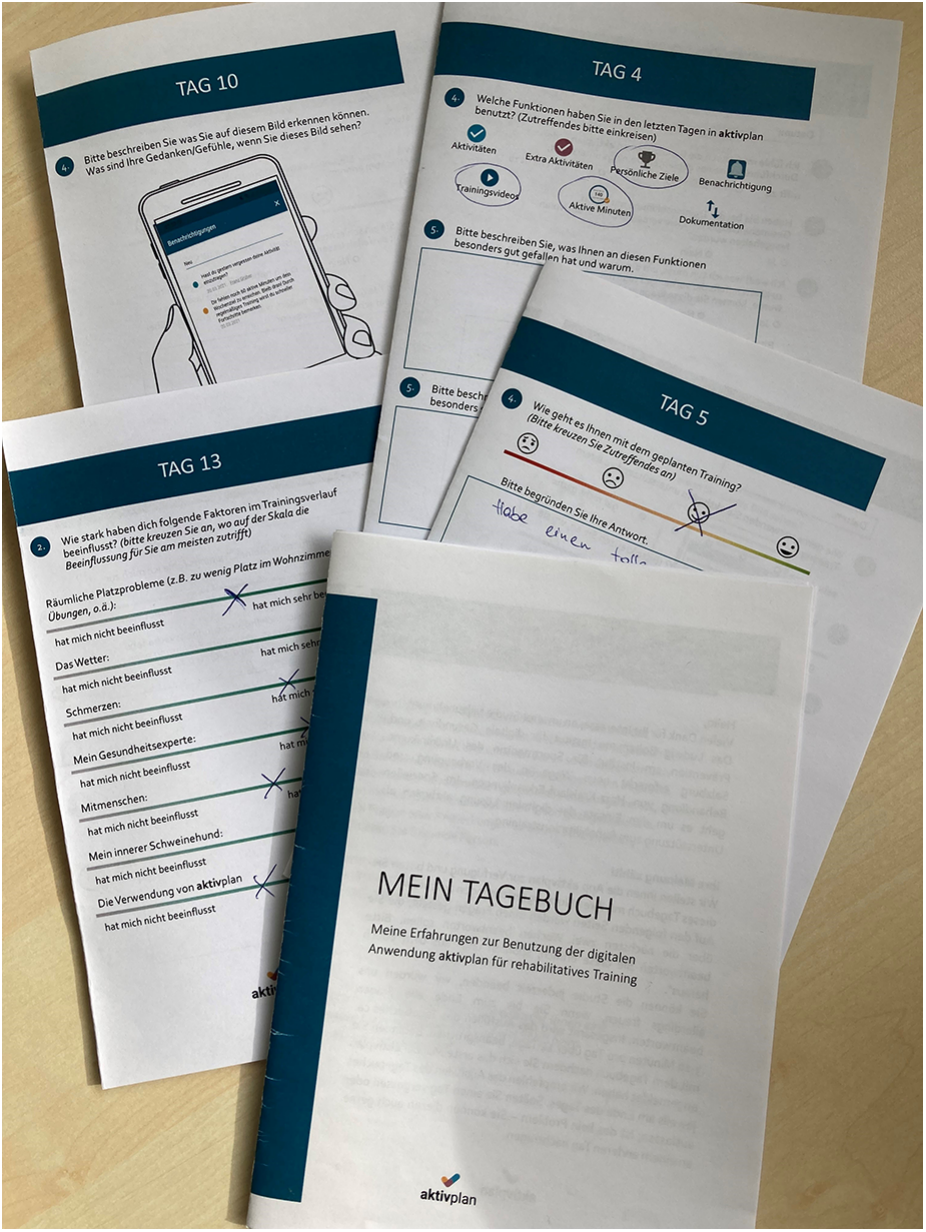
Exemplary pages of the diary given to persons with CVD during 14-day usage phase.

The **usage phase** was dedicated to app usage in daily life over a time-span of 14 days by persons with CVD. During this time, the person was asked to use the aktivplan app to perform, track and document their exercise training as planned with the healthcare professional in the PA consultation session. Frequency and types of training activities had been individually determined, depending on the person’s fitness level and state of health (as judged by the healthcare professional). In addition to using the app, the person with CVD was asked to complete a pen and paper diary (Figure 3) with questions about their physical activities, and impressions and usage experiences with the app (Section 2.1.3).

In the **final phase**, after 14 days of usage of the aktivplan app, the researcher conducted a final interview with the person with CVD. This interview focused on the person’s experiences and potential issues when using the aktivplan app in everyday life as well as suggestions for improvement (Section 2.1.2).

### 2.5 Data analysis

Audio and video data was transcribed by using a free version of the software Express Scribe Transcription. Data from the diaries was transferred to an Excel file by the reseracher. Diaries were completed well, i.e., there were no significant omissions, but there were variations in how detailed open-ended questions had been answered. For analysing the data, we used thematic analysis (25). This analysis approach is used to organize qualitative data sets by identifying different themes within the collected data. For our purpose, we aimed to structure and describe the collected data by revealing the prevalent themes regarding SDM in the context of establishing a PA plan with support of a digital tool. In a first step, we selected relevant data with regard to our research question, i.e., in how far SDM is manifested in the situated context when using a digital tool. Then, we assigned initial codes to the data and searched for themes based on the codes. We identified the following themes concerning the process of SDM in a tool-supported activity planning process: (1) Relations between persons with CVD, healthcare professionals and the digital tool, (2) Language as a strong influence, (3) Planning and goal setting, (4) Expectations before actual use. These themes are described in detail in Section 3.3.

## 3 Results

In this section, we describe participants demographics, results of the SDM rating scales and insights based on the thematic analysis.

### 3.1 Participants demographics

A total of seven male persons with CVD took part in the study. All of them were enrolled in a cardiac rehabilation programme at the affiliated rehabilitation facility. Our sample consisted of males only as there was only one woman training at the affiliated rehabilitation factility who would have fulfilled the inclusion criteria (as opposed to 23 males) at that point in time. Participants’ age ranged from 65 to 83, with a mean age of 71 (M=70,71; SD=6,18). Their educational background was mixed, with two participants holding a university degree, three having completed secondary school, and two having completed mandatory school. All of the participants regularly attended the affiliated facility for their rehabilitation training, which means they already knew the structures and the healthcare professionals. Patients’ experience of cardiac rehabilitation (i.e., the time since the first cardiac event) ranged from six months to ten years, with a mean value of six years (M=6,35; SD=3,64). With regard to previous experience with technology usage, three participants indicated that they were experienced in using apps, while four reported that they did not use apps in their everyday life, i.e., they were not experienced in the use of apps. Most of the participants already used digital support for training (n=5; smartwatch, pedometer, fitness app on mobile phone). All participants were from Austria and lived in Salzburg at the time the study was conducted.

A total of five healthcare professionals participated in the study, all working at the affiliated rehabilitation facility. Three of the participants were sports scientists by profession, one was conducting an internship (sports scientist in training), and one was a physician by profession. They were aged between 21 and 41 years, with a mean age of 34 years (M=33,80; SD=8,87).

### 3.2 SDM ratings via OPTION & RPAD

The mean score of OPTION was 17 (SD=7,26; 12 items ranging from 0–4; maximimum possible score = 48, with higher scores indicating greater level of SDM). The mean score of RPAD was 5,3 (SD=1,44; 9 items ranging from 0–1; maximimum possible score = 9, with higher scores indicating greater level of SDM). These mean scores indicate that the shared decision level of the dialogue could still be improved.

### 3.3 Aspects of SDM with a digital health tool

In the following, we point out how the use of a digital health tool for PA planning can be related to SDM. We identified the following themes around the process of SDM in a tool-supported activity planning process: (1) Relations between persons with CVD, healthcare professionals and the digital tool, (2) Language as a strong influence, (3) Planning and goal setting, (4) Expectations before actual use. Based on these themes, we describe different aspects that show the manifestation of SDM when using the aktivplan app.

#### 3.3.1 Relations between persons with CVD, healthcare professionals and the digital tool: interactional aspects

Before introducing the aktivplan app to the healthcare professionals and persons with CVD, the normal routine for conducting rehabilitation was to perform an exercise capacity assessment (ergometry) at the beginning of the rehabilitation. After the assessment, the healthcare professional and the person with CVD sat together. The healthcare professional discussed results and implications from the assessment with the person with CVD, and suggested which activities to perform at home. Depending on the healthcare professional, there was more or less emphasis on SDM. Then, by providing the aktivplan tool to the healthcare professional, a technical device was involved in this consultation situation. This device was used actively by most healthcare professionals as a **means for visually supporting the conversation**. Observational data showed that four out of five healthcare professionals explicitly used the computer monitor to explain functionalities of the app, in particular to show what the calendar overview looks like. The fifth healthcare professional did not use the computer monitor but explained the functionalities of the app in a verbal-descriptive way without demonstration (healthcare professionals could choose how they wished to explain the app to the person with CVD, i.e., there was no instruction given). For example, HP2 indicated: “*Right, now we have the training sessions on Tuesday and Thursday in the calendar [points to monitor]. And on Sunday we have added a 45-min walk*.”

Involving a technical device in a counseling process was also related to **technical flaws and challenges**, e.g., one professional (HP3) said “*…I have now created a user profile for you here on the computer*,” thereby rotating the monitor towards the person with CVD and causing a malfunction by that (display vanishes). In general, the situation of sitting together in a room, i.e., the shared physical presence of both person with CVD and healthcare professional, was experienced positively by the persons with CVD. The spatial arrangement of sitting together in front of a computer screen and deciding about the exercise training plan can be understood as a physical manifestation of sharing. Watching along on the computer screen was associated with being interesting, having a **learning effect and conveying transparency**. For example, P4 indicated: “*It is important to watch the screen, you have the feeling that it is transparent*.” Persons with CVD further stated that **interacting with healthcare professionals provided support and control** and was **perceived as motivating**. This was considered important for positive health behaviour change. P5 formulated this as follows: “*I needed the medical side, because otherwise I wouldn’t have done it. Because I rely on it completely…and then I want to stick to that*.” Similarly, P3 pointed out: “*Personally, it took me about three months to change my diet and the physiotherapist helped me tremendously. So for me, it was just the luck of having the physiotherapist and the family doctor*.” Personal relationships, in particular with healthcare professionals, turned out to be crucial for individuals’ motivation. In addition, healthcare professionals positively influenced health behaviour change of persons with CVD through knowledge provision and support.

#### 3.3.2 Language as a strong influence: conversational aspects

An important aspect in SDM was the use of appropriate language to emphasize that a joint decision would be made. The example of HP5’s introductory words nicely showed the **targeted involvement of persons with CVD through the use of appropriate language**: “*We will then go through the data protection, where you then agree and then I would ask questions about your sports history, what you are currently doing, what you have done in the past. And then I would develop a plan together with you, which you can then also see on your mobile phone, for the next four weeks. Is that ok for you?*” (HP5). Regarding such an involvement via language, the use of the word “we” by healthcare professionals was found to be regularly used, e.g., “*So now we’re going to plan this*” (HP4); “*…we look at where we are [at the moment] and then the goal where we want to go*” (HP3). Overall, four out of five healthcare professionals used the key word “*we*” multiple times. Related to that, the term “*together*” emerged in the conversations of two healthcare professionals. The **provision of support and assistance** by healthcare professionals was acknowledged by persons with CVD and can be interpreted as a manifestation of SDM. Patients positively mentioned having a point of contact for questions and the feeling that someone is there in case of insecurities or problems during their rehabilitation. Four out of five healthcare professionals explicitly stated that persons with CVD could ask any questions and offered their support. All participants asked questions during the process of establishing a training plan. Many of these dealt with the question of how to use the app at home (on their mobile phone), which data had to be entered and where, or what counted as a so-called extra-activity. SDM was addressed in the conversation via the language used as well as by actively offering assistance. However, none of the healthcare professionals explicitly mentioned the adoption of a SDM approach directly towards the persons with CVD. With regard to the usage of the app after the PA consultation session, persons with CVD often mentioned that monitoring and insight of healthcare professionals into their activities was desired as this enabled **control and support**, providing them a **feeling of safety and togetherness** (not being alone). For example, P5 stated “*For me it’s very important, I think it’s really good when I know that there’s a medical side that pays attention, that’s there for me. I feel safer there*.” Knowing that there was a future appointment with the healthcare professional further motivated the persons with CVD as a goal to work towards.

#### 3.3.3 Planning and goal-setting: decisional aspects

After data from the person with CVD (e.g., weight, height, maximal heart rate) was entered into the app and agreement on data usage and voluntary participation was given, the healthcare professional could freely decide about the further procedure. This should include the explanation of the app and its functionalities, the definition of the goals, and the creation of an exercise training plan. This part tended to be rather unstructured in the sense that every healthcare professional acted differently (e.g., some defined goals before the training plan and vice versa). For example, one healthcare professional did not explicitly mention the goal of the app. The other healthcare professionals mentioned the goals but did this in different ways, using terms such as “support,” “visualise data,” “planning,” “monitoring,” and “individual training.” Although there was a sequence of steps suggested by the provided materials, this was not necessarily adopted. The **lack of routine when working with the digital tool** was for example expressed in the following statement: “*Ah, I just have to follow these guidelines a bit so that I don’t forget anything*” (HP4).

Within the process of creating a training plan, talking about **routines and habits of persons with CVD** was important. Considering preferences and everyday routines of persons with CVD was a source of motivation for individuals and should be part of PA planning. PA was the topic most prominently addressed with regard to already established activity routines and preferences for specific sports. Hereby, going for a walk - with the dog, the partner or peers from cardiac rehabilitation - was the activity mentioned most often by persons with CVD. Next to that, football, hiking, and cycling were popular activities. In this context, persons with CVD often indicated **influences on their activity level**. In particular, pain and weather played a crucial role when it came to conducting a targeted activity. For example, P3 mentioned weather as a determinant of chosen activities: “*It’s relatively weather dependent [how active I am]…It’s more when the cycling season is over - November, December - that I do walking again, but when the weather is like this I prefer cycling*.” Similarly, P5 stated: “*My weekly schedule strongly adheres to the weather, whether I’m exercising alone or in a group*.”

P7 refered to pain as an inhibitor of activity: “*Yes, the time would be there and I had to put the brakes on because the pain in my back was so bad that I couldn’t do the exercises and then of course I didn’t do them. As it is now, I could certainly do certain exercises*.” Overall, we found that current habits and sports activities of persons with CVD were discussed, however, general preferences and potential new sport activities were only marginally addressed or not addressed at all by the healthcare professionals.

When planning activities, healthcare professionals entered activities from the rehabilation facility and activities already regularly performed by the persons with CVD into the app. The process of establishing a training plan required most of the conversation time between healthcare professional and person with CVD. Hereby we found a clear lead of healthcare professionals, having the longest talk time by far and persons with CVD being rather passive, having their main task in answering questions (e.g., regarding preferred days for planned activities or their pulse). Details of this process varied individually, meaning that some healthcare professionals asked persons with CVD much more than others. Participants experienced the process of planning the exercise training plan as positive. In particular, **integrating personal interests and social context in the training plan** motivated persons with CVD. The following statement illustrates the importance of (considering) the individual’s social context: “*My wife and I push each other…Have you already done that, I’m going to train now or let’s go for a walk. That is our community, we are already [active] together. We have been married for 60 years*” (P1). According to the persons with CVD, they mostly appreciated that the training plan was established together and tailored to their interests. Vice versa, not considering personal interests of persons with CVD was counterproductive as could be seen in the statement of P5, who indicated that he would change the plan afterwards as his interests with family were not considered.

Regarding the goals which were set for the persons with CVD, some healthcare professionals mentioned that they did not actively consider goals so far, but perceived **goals as motivation and incentive** for conducting activities. When it came to goal-setting by means of the app (dedicated functionality), we found that this was done differently by the healthcare professionals. Particularly, the definition and realisation of “goal” was diverse amongst heathcare professionals. Specifically, four healthcare professionals deliberately drew on personal interests and formulated goals based on before mentioned interests of persons with CVD. For example, based on the before stated interests and preferences, HP3 suggested the following: “*…we could set a target in the next few weeks with a 45 km bike ride*” (HP3). Goals were actively requested only by three of the five HPs. This could also related to the fact that persons with CVD sometimes had already formulated goals for themselves, e.g., P5 indicated that his goal was it to climb a specific mountain, to go all the way up there. He further pointed out: “*The goal is I want to go up and now I’m going up to half time and if I’m doing well then I’m going up. Thanks for the structure*.” In general, participants appreciated to have (more or less) individually set goals. However, two persons with CVD were sceptical about the feasibility of the set goals. The **perceived feasibility of a goal** was found to be crucial for an individual’s motivation to conduct the planned exercises and stick to the training plan, implying that unrealistic goals may have a negative influence on the individual’s adherence.

#### 3.3.4 Expectations before actual use: anticipatory aspects

Patients’ expectations towards a digital tool for PA planning were mainly related to the aspect of being **supported to regularly perform exercise**. For example, persons with CVD expected to (begin and) stick to being active by **having a structure** and plan which they have to follow. Those who had previously been active hoped to get back into former activity patterns. Further, a calender with planned activitities represented a **feedback mechanism** which mirrored and explicitly represented the performed behavior. For example, P1 states: “*Yes I think so [that the app is helpful]…so that you have a mirror in front of you*.” Similary, P4 stated that entering and looking up activities provided better control of oneself: “*Yes, probably [the app is helpful], because you should really enter something and look it up, and thus you control yourself even better*.” This structure which was provided by experts also concerns **realistic goal-setting**, i.e., having more realistic training goals (compared to those which are defined by oneself): “*[I expect from aktivplan] that you really keep to the training goals, because often you are a bit too optimistic yourself*” (P3). We also found **concerns regarding app usage** before the launch. This initial scepticism in persons with CVD was mainly due to a lack of skills in using digital applications. Some mentioned that this is difficult to change/learn when older. They also reported fear of being overwhelmed, as a lot of information was expected to be processed at the beginning for them. Further, one participant was afraid that using the app would mean a lot of effort for him: “*[In the app I] have to choose what I do and that’s a certain amount of work*” (P3). Within the present study, the **importance of the enrolment and introduction process** of the app was expected to be significant. Persons with CVD indicated that getting started is important for motivation to continue using the app. The first contact was considered to be crucial, e.g., the registration process and the activation of the account. From the persons with CVD side, there was the expectation that there was information and guidance on what to do and how to proceed with the app. **Motivation** was another issue regularly mentioned with regard to expectations of the app. One source of motivation was the on-site training at the rehabilitation facility. This was linked to training without fear, training in a group with other persons with CVD, and under expert supervision (P2). The expectation that activities can be viewed by healthcare professionals was considered as another source of motivation (i.e., by having the feeling of being controlled). Besides supervision and training together with other persons with CVD, another source of motivation was attributed to pursuing personal interests (walking with the dog, hiking to see nature).

When interviewing health experts about their expectations and attitudes towards the app, the app was seen as a **connection between healthcare professionals and persons with CVD**, providing them the opportunity to guide and stimulate persons with CVD not only at the rehabilitation facility but also in their everyday life: “*They go home and do things, they know they should exercise and so on. And so I intervene directly and say ok, what does your everyday life look like, when would you have time to do something and I can give him my guidance, my instructions for why don’t you take a look at this, I can give him ideas where he might say, ok, I wouldn’t have thought of that idea*” (HP2). Such a bridging function seems to be of particular value as this integrates the “home,” i.e., the everyday life of persons with CVD. This was not the case before, meaning that activities at home were not actively considered in the planning of exercise training. Thus, it was seen as a new approach compared to the “traditional” rehabilitation practice at the facility. In the words of HP1: “*…but what he did at home, we don’t look at that. And I think that would be a starting point with gadgets…It’s a different approach…. monitoring allows checking [the activities of person with CVD] and also keeping them motivated*.” Healthcare professionals expected the app to be **patient-oriented, intuitive and simple**. It should actively involve persons with CVD and provide a **concretisation and specification of training activities** to them, and promote a more detailed discussion with persons with CVD. This was expected to lead to a more sustainable training and better outcome (i.e., physical performance) at the end. It was further expected to give healthcare professionals **insight into the performed activities** of the persons with CVD. Checking later what the person with CVD had done and actively dealing with activity from the person was thought to be an important part of the app usage. In relation to that, data protection issues were expected to be handled by the tool.

## 4 Discussion

### 4.1 SDM supportive design in digital health apps

In our study, we identified four aspects concerning the process of SDM in a tool-supported PA planning process which should be considered when integrating SDM in digital health tools: (1) interactional aspects of SDM, dealing with relations between patients, healthcare professionals and the digital tool; (2) conversational aspects, emphasising the influence of language when a digital tool is part of the SDM process; (3) decisional aspects, focusing on the process of planning and goal-setting supported by a digital tool; and (4) anticipatory aspects, putting emphasis on the expectations before actual use of a digital tool in relation to its users and their relationship (i.e., the relationship between a healthcare professional and a person with CVD). Additionally, data from the OPTION and RPAD scales demonstrated potential for further improvement regarding the content and quality of the SDM dialogue, and interview and observational data highlighted the need for more structured goal-setting and more explicit consideration of preferences and routines.

Based on observed processes, expectations, and feedback regarding SDM, we formulate implications for design. In particular, we suggest how digital tools can support SDM. Our suggestions are based on the use case of planning PA for persons with CVD; however, we assume that they can also be useful on a more general level. As we consider SDM as a cyclical activity in relation to chronic diseases, we mapped our findings to temporal phases when SDM can be adopted. Table 1 provides an overview on how SDM can be better integrated in digital health apps.

**Table 1:** Overview on SDM supportive design in digital health apps.

When *point in time regarding consultation*	What *actions that can be taken*	Why *purpose regarding SDM*	How *digital implementation of actions*
Before PA consultation session	Provide information	Preparation	Dedicated platform for patients providing information sources
	Trigger reflection		Dedicated platform for patients eliciting routines, habits and preferences
	Provide data		Dedicated data base/platform for health professionals where (already) available data from patients is accessible
During PA consultation session	Create collaborative atmosphere	Decision making	Step-by-step guidance (“digital stepper”)
	Check knowledge and inform		
	Discuss data		
	Set goals		
	Define exercise plan		
	Inform about next encounter		
After PA consultation session	Offer resources	Long-term adherence	Activity monitoring
	Ensure contact person		Reminder messages
	Offer additional support		Time plan (further appointments)
			Online resources

In the first phase, i.e., before the actual PA consultation session, SDM is intended to prepare both the person with CVD and the healthcare professional for the upcoming encounter. According to Carmona et al. (26), this phase is about reflecting on what matters to them, what they hope will happen as a result of the discussion, and what questions they would like to ask. In line with the description above, our data pointed out the importance of addressing concerns and expectations of persons with CVD to optimally prepare them and avoid false assumptions. Expectations that persons with CVD have before getting to know the app are important to consider, so that negative associations, doubts and fears can be intercepted beforehand by adequate preparation and information. Based on our findings, we suggest three thematic blocks. To optimally prepare persons with CVD, we suggest (1) information provision (e.g., about SDM, CVD or digital tools) as well as (2) reflection triggering (e.g., about routines, habits and preferences). To optimally prepare healthcare professionals, we suggest (3) data provision (e.g., patient data such as personal data or health data from clinical assessments, data collected from wearables, or data regarding the preferences and habits of persons with CVD. These blocks could be provided and elicited on a dedicated platform for persons with CVD (e.g., a website). Similarly, a dedicated data base/platform for healthcare professionals could give them the opportunity to optimally prepare for the upcoming consultation and could further provide some time-saving.

In the second phase, i.e., during the PA consultation session, decisions should be made. With regard to our use case, these decisions should result in set goals and an exercise training plan. In our study, we found a strong need for more structured guidance for healthcare professionals. Based on previous work of Carmona et al. (26) and Elwyn et al. (27), aligned with results from our use case investigation, we suggest that the following issues should be addressed in a face-to-face appointment in which PA planning takes place: (1) create a collaborative atmosphere to make sure that persons with CVD or service users understand that they can participate as much as they want and feel encouraged, (2) check knowledge and inform about the digital tool, data usage and privacy issues, (3) discuss recorded data (e.g. from clinical assessments or activity records) to show where the person with CVD can actively contribute/improve, (4) set goals and provide good goal-setting support, (5) define an exercise training plan as a basis for long-term PA, and (6) inform about the next encounter and the time in-between to provide guidance on how to go on (in case of questions, changes,…). To use the potential of digital tools and provide structured guidance within the SDM process, we suggest to implement a step-by-step guidance (“stepper”). This stepper guides the healthcare professional throughout the conversation and assures that all parts are discussed. The stepper is thought to provide a rough overview which can be “zoomed in” on two levels: when clicking on a dedicated item, a more detailed description is provided (level one); by clicking a second time on a dedicated item, examples on how to phrase a specific aspect of the conversation are provided (level two). This should offer healthcare professionals flexible support in remembering and articulating key statements that are essential for a communication style that facilitates SDM.

In the third phase, i.e., after the PA consultation session, emphasizing follow up meetings is important (especially in the case of chronic diseases) with regard to long-term adherence. Based on previous work (e.g., (26, 27)) as well as our own insights, we suggest to take the following actions: (1) Offer people resources to help them understand what was discussed and agreed, (2) ensure that information provided after discussions includes details of who to contact for any further questions, (3) offer additional support to people who are likely to need extra help to engage in SDM. Our results showed the importance of tracking activities (i.e, by automated or manual reporting). This induces a feeling of being monitored in persons with CVD and in turn motivates them to be active, and at the same time provides valuable information to the healthcare professionals. Further, communication channels play a crucial role in order to make the SDM “sustainable.” Providing means of communication and prospects of further consultations are therefore considered important factors towards persons’ with CVD adherence. As more (physical) meetings increase the adherence but are often not realistic due to the high time effort for healthcare professionals, a good balance should be found here. Besides, alternative communication channels could be implemented. For example, an automated reminder message (e.g., reminding about a planned activity) with a picture of the associated healthcare professional could be thought of as low-effort compensation. Finally, the provision of online information material can be beneficial for long-term adherence.

### 4.2 Challenges & opportunities

Although SDM is getting increasingly established in clinical practice, the application of SDM can still be improved. As stated in the introduction, SDM bears potential for empowering persons with CVD and supporting long-term behaviour change (16–18). A systematic attempt to bring together SDM and behaviour change was made by Bonneux et al. (15). In our digital tool we found the stated principles to be integrated. However, as seen in the present study, issues such as goals, habits and preferences are often discussed on a general level. Here, improvements towards a more structured process could be beneficial, guiding the conversation towards a more detailed and individual level. Based on our data, we see the most pressing issues to be tackled as follows: We made the observation that **goals** are defined very differently and in an unstructured way. From the viewpoint of a person with CVD, perceived feasibility of a goal was found to be crucial for the motivation to conduct the planned exercises and stick to the training plan. This corresponds to Rutjes et al. (12), highligthing the importance of specific and achievable goals to stay motivated and increase self-efficacy. To improve and structure the process of goal-setting, we suggest a digital step-by-step guide as described in Section 4.1. In addition to goals, **preferences and routines** need explicit consideration. This is in line with Rutjes et al., who points out that “the more specific and tailored the advice, the more likely clients are to adhere to it” (12, p. 5). To come to individually optimized decisions - in our case personalized training plans - we suggest a dedicated platform which supports persons with CVD to reflect on preferences and routines before the actual PA consultation session takes place (Section 4.1). Finally, **time efforts** are crucial for healthcare professionals. To reduce time effort, we suggest to collect data about the person with CVD (e.g., personal or health related data, preferences and routines) before the actual PA consultation session and provide it to the healthcare professional (so that elicitation during the session is not necessary any more and the healthcare professional can prepare in an optimal way).

We further argue that successful and sustainable SDM has to be implemented on an organisational level. To be successully and sustainably implemented in clinical practice, SDM has to be seen not only on a micro-perspective but also from a macro-perspective. In our study, healthcare professionals pointed out the demand of acknowledging and supporting SDM on an organisational level. For example, a potential shift of healthcare professionals’ time resources as well as onboarding and learning how to use a digital device for establishing a shared decision has to be considered (from higher level management). This is in line with Carmona et al. (26), who states that SDM is more likely to become standard practice in organisations when it is led from the highest levels of the organisation.

Overall, we argue that digital tools hold great potential for facilitating and shortening processes and thus offer invaluable opportunities for supporting SDM in clinical practice. For example, step-by-step guidance and in-situ phrasing examples, visualizations of progress and goals, and adequate preparation of persons with CVD and healthcare professionals can be effective means for that.

## 5 Conclusion

As Elwyn et al. stated, *“new systems will be required to appropriately reward truly patient centered practice”* (27, p. 1366). Such a practice centered on SDM is thought to foster sustainable adherence to physical exercise (18) and thus helps to bridge the gap between on-site and remote exercise training in cardiac rehabilitation (16). In the context of a field study, we investigated how SDM was already applied and manifested when using a digital tool for heart-healthy PA planning in clinical practice. Based on that, we have identified when additional support would be beneficial within the process of striving for a shared decision and have pointed out the potential of digital tools that can be used to better support SDM.

## Data Availability

The raw data supporting the conclusions of this article will be made available by the authors, without undue reservation.
