# Supplementary material for: Investigating shared decision-making during the use of a digital health tool for physical activity planning in cardiac rehabilitation
Source: Front Digit Health. 2024 Jan 4;5:1324488. doi: 10.3389/fdgth.2023.1324488 (PMC10794499; doi:10.3389/fdgth.2023.1324488)
Supplement: Supplementary file 1 [file Datasheet1.pdf]

Patient inclusion criteria:

- Adults (18 years and older)
- Cardiovascular disease (CVD) with current or previous participation in medical exercise therapy, i.e.., patients in phase III or IV of cardiac rehabilitation according to the Austrian healthcare system.
- Technology affinity: openness to new technologies and use of smartphone in everyday life but no special previous technical knowledge necessary.
- Medical examination or clearance for participation in medical training therapy in the last 3 months
- Signed informed consent form

Patient exclusion criteria:

- Medical contraindications to the performance test, or to regular physical exercise.
- Limited German language skills
